# Current research and future directions for realizing the ideal One-Health approach: A summary of key-informant interviews in Japan and a literature review

**DOI:** 10.1016/j.onehlt.2022.100468

**Published:** 2022-12-05

**Authors:** Kiyohiko Andoh, Arata Hidano, Yoshiko Sakamoto, Kotaro Sawai, Nobuo Arai, Yuto Suda, Junki Mine, Takehiko Oka

**Affiliations:** a National Institute of Animal Health, National Agriculture and Food Research Organization, 3-1-5 Kannondai, Tsukuba, Ibaraki 305-0856, Japan; b Communicable Diseases Policy Research Group, Department of Global Health and Development, Faculty of Public Health and Policy, London School of Hygiene and Tropical Medicine, United Kingdom; c National Institute for Environmental Studies, 16-2 Onogawa, Tsukuba, Ibaraki, 305-8506, Japan; d World Fusion Co., Ltd., 1-38-12 Nihonbashi Kakigara-cho, Yusho-kaikann 2F, Chuo-ku, Tokyo, 103-0014, Japan

**Keywords:** Pandemic, Metadata, Wildlife, Planetary health, Cross-sector, Interdisciplinary research

## Abstract

The COVID-19 pandemic has highlighted the importance of the One Health (OH) approach, which considers the health of humans, animals, and the environment in preventing future pandemics. A wide range of sustainable interdisciplinary collaborations are required to truly fulfill the purpose of the OH approach. It is well-recognized, however, that such collaborations are challenging. In this study, we undertook key-informant interviews with a panel of stakeholders from Japan to identify their perceived needs and challenges related to OH research. This panel included scientists, government officials, journalists, and industry stakeholders. By combining a thematic analysis of these interviews and a literature review, we summarized two key themes pertinent to the effective implementation of OH research: types of required research and systems to support that research. As a technological issue, interviewees suggested the importance of research and development of methodologies that can promote the integration and collaboration of research fields that are currently fragmented. An example of such a methodology would allow researchers to obtain high-resolution metadata (e.g. ecological and wildlife data) with high throughput and then maximize the use of the obtained metadata in research, such as in environmental DNA analysis, database construction, or the use of computational algorithms to find novel viral genomes. In terms of systems surrounding OH research, some interviewees stressed the importance of creating a sustainable research system, such as one that has continuous budget support and allows researchers to pursue their academic careers and interests. These perceptions and challenges held by Japanese stakeholders may be common to others around the world. We hope this review will encourage more researchers and others to work together to create a resilient society against future pandemics.

## Highlights

• Interviews were conducted with Japanese stakeholders in the One Health (OH) approach.
• Experts provided their views on the opportunities and challenges of OH research.
• R&D methodologies accelerating interdisciplinary research should be strengthened.
• Continuous budget support and facilitation of academic careers are necessary.

## 1 Introduction

How can we prepare for and mitigate the impact of future pandemics? The COVID-19 pandemic has highlighted not only the enormous damage that emerging diseases can cause to individuals and society but also the urgency for society to identify areas of actionable science that will enhance our pandemic preparedness. One such arena is improved surveillance systems of humans, livestock, and wildlife [1]. Given that some recent pandemics have an origin in wildlife, scientists have called for a One Health (OH) approach to better understand the spillover risk from wildlife populations [1,2]. The importance of wildlife surveillance has been stressed since the early 2000s [3], but this call for action has not spurred sufficient interdisciplinary collaboration in the past two decades.

While surveillance is critical, there are also many challenges in implementing surveillance in human, livestock, and wildlife populations, especially in low- and middle-income countries. A fundamental lack of resources means that the surveillance needs to be narrow, prioritizing subsets of the population. This need has spurred major efforts to predict the spillover risk of unknown pathogens. For example, the United States Agency for International Development Emerging Pandemic Threats PREDICT program has discovered approximately 1000 novel viruses and has attempted to rank the spillover risk of these viruses using risk factors identified through literature reviews and from expert opinions [4]. Some prominent virologists, however, have been skeptical about the feasibility of such predictions [5,6], arguing that this approach suffers from multiple problems. The first problem is the lack of scalability. Newly discovered viruses require genetic sequencing and laboratory analyses to assess their pathogenesis and transmissibility in humans. Nevertheless, the number of undiscovered viruses in nature is estimated to be tremendous, suggesting that substantial resources and time would be required to complete this task. The evolution of viruses also undermines the usefulness of these data, which have been collected at a single point in time in the past. The second problem is the significant knowledge gap about ecological factors that facilitate spillover and transmission of emerging microorganisms [7]. Previous spillover events suggest that sustained transmission of emerging viruses in new hosts is the exception rather than rule, even though the viruses have genetic characteristics required to infect specific species. Spillover and sustained onward transmission probably require specific ecological and epidemiological conditions for different viruses [[7], [8], [9]], and these remain poorly understood.

Some scientists argue that technological innovation may overcome these challenges and enable zoonotic risk prediction [10]. Although we acknowledge that such predictions are one of the key desired scientific outputs, it is important to recognize that these aforementioned challenges themselves highlight that previous OH studies were often concerned solely with microbiological aspects of zoonoses. We contend this focus needs to shift towards a more integrated approach in which complex interactions between ecology, epidemiology, biology, and evolution are studied (e.g. evolutionary epidemiology [11]). Currently, we have little understanding of the mechanism behind spillover transmission from wildlife even for known diseases, such as Ebola and severe fever with thrombocytopenia syndrome (SFTS). Moreover, disease dynamics may change in response to alterations in ecology induced by factors such as climate change, biodiversity loss, and land-use changes [12]. Hence, effective control of these known diseases requires a deeper understanding about how the ecology of host wildlife species and environments shape disease dynamics and evolution. We believe this understanding will also facilitate better risk predictions of unknown zoonoses.

These knowledge gaps can be filled only by OH studies that are committed to gathering metadata associated with microorganisms. These studies require truly interdisciplinary collaborations that enable researchers to identify what kind of data are required to achieve the objectives mentioned above and to determine how the data should be collected. However, as highlighted in previous studies, many challenges exist in initiating, designing, and implementing truly interdisciplinary OH studies [13]. These challenges include a lack of sustainable funding, underrepresentation of some actors, unsustained efforts, difficulty in promoting sustained interdisciplinary collaborations, and institutional and systemic fragmentation. Without solving these challenges, studies may be driven by stakeholders from limited disciplines and risk having insufficient access to data sources and analyses.

While momentum for OH approach is growing in Japan, interdisciplinary collaboration is still limited. To identify specific issues for accelerating interdisciplinary collaboration, we undertook key-informant interviews with stakeholders from a wide range of disciplines and sectors in Japan. We asked each interviewee how we can build a society that will be resilient to disease outbreaks and what needs to be done to accomplish this task, as well as how they could contribute to it. We then conducted a thematic analysis of these interviews and a literature review and summarized obtained insight into two thematic areas: types of required research and development (R&D) and the systems needed to support them.

## 2 Methods

From September to December 2020, we conducted an initial literature review of the challenges and opportunities related to OH research, the findings from which guided our selection of disciplines and individuals for interview. Key-informant interviews with stakeholders in Japan were conducted in January to July 2021 (interviewees are listed in Supplemental Table 1). These stakeholders included not only scientists who have been actively involved in OH studies but also others who are highly relevant but not always involved in OH studies, such as data management specialists, artificial intelligence (AI) scientists, journalists, and representatives of industry and government, and we asked each interviewee about their perceived needs, challenges, and opportunities related to OH research and discussed key themes pertinent to the effective implementation of OH research (see Supplementary material). Each interview was transcribed and analyzed through an iterative process of open/selective coding with a constant comparison with findings from our literature review. These results were summarized into two overarching themes: types of required R&D and the systems needed to support this R&D. This study was judged to be low risk by National Institute of Animal Health and JST Moonshot R&D –MILLENNIA Program, Japan, and further ethics approval was not required. Verbal consents were obtained from all the participants to participate in this study. Upon the completion of drafting the manuscript, a written consent was obtained from the participants to use their quotes after they have read the manuscript. The participants were given an option to be anonymized or reveal their names.

## 3 Results

### 3.1 Required R&D for realizing the ideal OH approach

#### 3.1.1 Importance of conducting interdisciplinary studies

Many current surveillance systems need to start collecting additional metadata, such as data about wildlife ecology or vectors and environmental factors, because these are crucial for understanding pandemic risk [12]. Wildlife ecology affects viral dynamics in nature, and the habitat of animals drastically changes depending on soil and vegetation conditions. The distribution of vectors such as arthropods or blood-sucking insects is also affected by the relationship between the environment and animals, and the contact frequency between wildlife and livestock animals depends on land utilization. Analysis of such metadata requires an interdisciplinary approach integrating disciplines such as virology, bacteriology, parasitology, molecular biology, immunology, population genetics, ecology, and epidemiology. Our key-informant interviews highlighted the need for technologies for collecting, connecting, and analyzing all related information, including the genomes of pathogens, wildlife ecology, local ecosystems, and seroprevalence of specific pathogens in wildlife or livestock animals, to accelerate interdisciplinary research.

#### 3.1.2 Technologies for collecting information about pathogens in nature

A new methodology for efficiently collecting viral genomes from field samples is required because surveillance for unknown viruses harbored by wildlife is an essential and effective countermeasure against future pandemics [1,14]. Recent advances in high-throughput sequencing (HTS) technology have dramatically improved genome analysis in terms of both quality and quantity; however, our key-informant interviews indicated that detecting a novel virus from nature is still a challenge. Viral genomes are extremely small and diverse compared to the host genome, and enrichment and separation methods are required for different viral species before HTS technology can be applied. In fact, the PREDICT program employed a conventional PCR method and not HTS, despite the large-scale nature of the research [14]. Recently, a novel technique called Fragmented and primer Ligated dsRNA Sequencing (FLDS) was developed by Urayama et al. [15]. The FLDS method can extract virus-derived double-stranded RNA, which is produced during the replication process of an RNA virus, making it easy to enrich and discover novel viral genomes. Thus, advanced technologies that allow the efficient isolation of viral genomes would foster breakthrough improvements in collecting viral genome information with far greater efficiency than is currently possible.

In addition, the scientists we interviewed mentioned that a new methodology needs to be developed that can identify completely unknown viral genomes from enormous amounts of metagenome data. Currently, most viral sequences in the metagenome data are identified based on their similarities to known viral species. For example, analyzing sequence similarity using the Basic Local Alignment Search Tool (BLAST) is a common method to detect unknown viral genomes. However, this method may fail to detect completely unknown viruses. To overcome this problem, Hie et al. [16] reported a method using natural language processing, which can identify a hidden viral genome by considering the viral sequence as a language and interpreting its grammar and meaning. This research area is underexplored and one of the interviewees mentioned that there are also ample opportunities to apply existing methods such as hidden Markov models [17] to identify hidden viral genomes.

The scientists interviewed also emphasized that isolation of infectious viruses is a prerequisite for determining the characteristics of viruses; therefore, we need innovative technologies that can efficiently culture novel viruses. For example, isolated infectious viruses are required for evaluating their host range, tissue tropism, virulence, and the efficacy of antiviral drugs, but it remains difficult to isolate an infectious virus with high throughput because cell culture techniques are laborious and time-consuming. Therefore, isolating viruses in a timely manner is one of the primary bottlenecks in interdisciplinary research. Recently, human induced pluripotent stem cells and human-derived organoids have been used to cultivate viruses that are hard to maintain in vitro [18,19], which can address this bottleneck. Further developments in reverse genetics technology may allow us to bypass this issue by artificially creating infectious viruses from a viral genome sequence.

#### 3.1.3 Evaluating viral dynamics in nature

Knowledge of viral dynamics in nature is fundamental for effective disease prevention and control. The presence of multiple host animal species in nature may hamper the acquisition of this knowledge. For example, in the COVID-19 pandemic, the true transmission route and missing link between reservoirs remain unsolved [20]. The scientists we interviewed suggested that such knowledge gaps can be filled through continuous surveillance by an interdisciplinary consortium; in Japan, SFTS is a prime example of a disease that warrants such surveillance.

SFTS is an emerging infectious disease that was first reported in China, and a total of 402 cases were identified in Japan between 4 March 2013 and 31 March 2019 [21]. SFTS is caused by the SFTS virus (SFTSV), which is maintained and circulated among ticks and other wildlife [[22], [23], [24]]. Several research teams in Japan, some members of which were included in our interviews, have conducted surveillance in humans, animals, and ticks to identify the dynamics of SFTSV and the risk of human infections. One study reported that the seroprevalence of SFTSV antibodies in deer exceeds 40% [25]. SFTS cases in Japan have mainly been reported in western regions where the seroprevalence of SFTSV in deer was greater than that in other areas, suggesting a positive correlation between the seroprevalence in deer and the number of human cases [25]. On the other hand, surveillance of SFTSV in ticks has shown that SFTSV is widely distributed across Japan, including in regions where no cases of SFTS have yet been reported [25,26]. The apparent disparity between the regions where human cases have occurred and the distribution of the virus suggests that other ecological factors may affect the contact frequency between humans, ticks, and deer or other wildlife. To predict the spillover risk to humans, these factors should be identified by detecting the SFTS virus in nature, conducting serosurveys in wildlife, and monitoring the ecology and environmental factors in each region. Accumulating such efforts is crucial to showcase the feasibility of predictions of zoonosis risk in the future. One of the scientists interviewed also pointed out that serosurveys of potentially susceptible livestock as sentinels [[27], [28], [29]] may indicate the extent to which a virus has spread around the human population.

Because any animal species may be a potential reservoir of unknown pathogens, the interviewees agreed that it is valuable to monitor the ecology of as many wildlife species as possible. However, they also pointed out that such monitoring is not feasible with the methods currently available, such as conducting long-term field observations or using fixed-point cameras, both of which are laborious and time-consuming. Hence, it is crucial to develop new monitoring technologies with high throughput and high resolution. For example, an environmental DNA (eDNA) and DNA barcoding technique could be useful for obtaining ecological information. Because eDNA comprises a range of substances from various origins, such as tissue fragments and feces dropped from organisms living in a habitat, HTS of eDNA would provide an enormous amount of information on the ecology of wildlife in a given area (e.g. distributions of habitats, numbers of individuals, and biomass of organisms) with a higher throughput, greater scale, and higher resolution than existing techniques [30]. The eDNA technique has been already applied to the collection of ecological information and to the study of infectious diseases [31,32]. These attempts might contribute to building an ideal risk management system, but some scientists have emphasized the limitations of eDNA technology. eDNA has mainly been used to monitor aquatic ecosystems and rarely been used in terrestrial ecosystems [33]. Furthermore, precise information such as age, sex, and blood relationship cannot be obtained. However, if eDNA technology becomes more applicable to terrestrial animals, and if improvements are made in terms of collecting more precise information, it should strongly promote interdisciplinary research across zoonoses and ecology.

To fully utilize the eDNA technique in zoonosis research, the experts interviewed mentioned that the genomic data of wildlife needs to be expanded. The current genome database does not contain all information required to identify all animal species. For example, while bats are considered to be natural hosts of several notorious viruses, only a limited number of bat species have had their genomes determined [34].

An improved genome database might also be useful when conducting biological experiments to characterize novel viruses. If the genomic information of all animals could be deposited in a database, all candidate receptors of novel viruses that exist in animals could be examined in vitro (e.g. by creating recombinant receptors and analyzing their affinity against viral ligands) or in silico (e.g. by predicting the structure of receptors and conducting docking simulations).

#### 3.1.4 Future prospects of interdisciplinary research in infectious diseases, epidemiology, ecology, and computer science

While recent major developments in computer science such as AI should be highly beneficial for research on zoonoses [10], these technologies are not yet readily applicable. For example, current AI technology requires a large amount of high-quality data as a training input, but both the quantity and quality of data available in current zoonosis research do not meet this requirement. An informatician we interviewed said that, “Zoonosis research is attractive, but only limited data is available to utilize our expertise”. Additionally, several interviewees argued that ontology and annotation are inconsistent and incomplete across different research fields, which also hinders the use of AI technology. Therefore, we urgently need to develop a standardized format for zoonosis data as well to reformat existing data.

Another requirement highlighted during the interviews was that the collected metadata must also be adequately linked and curated. Such linkage systems need to be well developed before we accumulate a huge amount of diverse data, such as genome sequences, ecological numeric data, and descriptive documents. One scientist said that resource description frameworks can be a useful methodology that connects several different types of data in a public network. The integration of multilayered data on ecosystems, soil, vegetation, climate, and viral metagenomes may enable researchers to conduct analyses using AI techniques and identify hidden associations between virus dynamics and ecosystems that are critical for pandemic prevention.

Other AI technologies such as those developed for image recognition algorithms and large data-driven research projects are also highly relevant for the research topics described in the above sections. Several interviewees said that image recognition algorithms may also be applicable for obtaining ecological data of wildlife in combination with camera traps and remote-sensing devices. Additionally, current popular topics in AI research, such as explainable, unsupervised learning and context-driven AI, may also be applicable to risk-management systems. For example, explainable and context-driven AI may be able to predict and explain a risk factor of future pandemics based on integrated information from descriptive documents, such as epidemiological reports and ecological metadata. Alternatively, AI may be able to predict a mutation pattern of a viral genome and consequent changes of receptor affinity based on the viral and animal genome database. Thus, these data-driven applications might be useful in decision-making and policy implementation as well as for scientific research [10].

Taken together, the interviews with experts in computer science highlighted the R&D required for improving interdisciplinary collaboration among zoonosis researchers and computer scientists. Continuous R&D can help fill the knowledge gap through interdisciplinary research and make it possible to predict the risk of pandemics, although perfect predictions may still be impossible.

### 3.2 Creating a sustainable system to control zoonoses

#### 3.2.1 Sustained and continuing zoonosis research

A common view among many interviewees was that securing long-term budgets is crucial for continuous zoonosis research. Although the importance of measures against outbreaks of unknown zoonoses is recognized, it is difficult to evaluate the monetary value generated by such activities, and this has undeniably limited the level of R&D. The COVID-19 pandemic has highlighted once again that research investment in infectious diseases is an extremely important security issue. Dobson et al. [35] calculated that the cost of 10 years of pandemic prevention research would amount to just 2% of the economic losses incurred by the COVID-19 pandemic. Thus, continued investment in preventive R&D is wholly rational in the long term. The top priority for some countries, including Japan, is therefore to gain acceptance from relevant stakeholders and the public so that they can invest in research and basic technologies related to infectious diseases as a national security policy. This will in turn strengthen countries' capacity to deal with national and international crises in a future pandemic.

One interviewee argued that it is crucial to maintain the motivation of researchers, and hence research topics should be chosen to meet the intellectual and academic interests of all the researchers involved. It is neither sustainable nor ethical to request the voluntary cooperation of researchers based entirely on goodwill, so we need to build a system in which everyone participating in a project or consortium can conduct research towards a shared goal while maximizing their own benefits.

#### 3.2.2 Social and economic issues: Stopping destruction of the natural environment to prevent future pandemics

There is a complex interplay of factors behind the spillover of zoonoses from wildlife to humans. A United Nations Environment Programme report [36] states that various social activities of humans are a major factor that is potentially responsible for spillover. Some of these activities are pressing issues, particularly in developing countries, including increased consumption of animal protein, the expansion of unsustainable agriculture, increased trapping and use of wildlife, and changes in the food supply chain. Other global factors, such as climate change, natural resource issues, and increased international human movement, are also relevant in the emergence and spread of zoonoses. Our interview analysis strongly indicated that not only scientific R&D but also research to overcome social and economic challenges is needed.

In the field of economics, the Dasgupta Review [37] contains many important recommendations to enable sustainable zoonoses management. Dasgupta considers biodiversity and its inherent associated ecosystem services as natural capital on which the economy is built. The review proposes the use of an inclusive measure of wealth, rather than gross domestic product to allow us to measure the well-being of current and future generations by considering all assets, including natural ones. Environmental degradation may contribute to pandemics such as COVID-19, which affect human economic activity. We therefore need a holistic approach that seeks to resolve environmental problems to reduce the risk of future outbreaks of zoonoses.

Both our interviews and literature survey [36,37] suggest that institutional reforms are crucial to sustain natural capital. We reason that we need a new measure of value that is mindful not only of traditional commercial value in terms of money, but also of environmental impact and sustainable development. As an initial goal, each country should aim to reduce investments in projects that degrade nature or involve its unsustainable use; facilitating this goal may well give rise to industrial re-structuring.

The management of global public health requires transnational institutions. Given that pandemics can affect all countries, global health initiatives should provide greater support for countries that are considered hot spots. A simulation study that combined data on infectious diseases with environmental factors suggested a higher risk of zoonotic disease emergence for areas with broadleaf evergreen forests, mammalian species richness, and significant changes in land use [12]. The same study suggested that all these environmental factors may also increase the opportunities for contact between humans and wildlife, which can lead to spillover of unknown pathogens into human populations. Our interviewees emphasized that no single field surveillance can cover regions across the entire world to search for unknown pathogens. Cooperation across countries and institutions are therefore essential. Systematic capacity-building will maximize the effectiveness of such global cooperation.

#### 3.2.3 Ethical, legal, and social issues

Several stakeholders who have participated in zoonosis surveillance in Japan highlighted the need to reform the vertical administrative system within authorities relevant to human, animal, and environmental health, as well as the laws, regulations, and systems that have hindered research and information-sharing [13]. For example, sampling of wildlife requires various administrative procedures, which can be a barrier to an effective response and implementation of control measures against zoonoses. In the future, a new administrative system, such as a department that collectively manages zoonosis projects, may be required to maximize the efficiency of zoonotic research and the efficacy of infectious disease control.

The interviewees who are currently involved in the fields of epidemiology, policy making, and mass communication said that a great number of issues remain to be addressed in terms of handling information such as the genetic sequences of pathogens. For example, in Japan, there is resistance to establishing a nationwide system for banking samples from livestock. Although it is well-recognized that such systems are useful for epidemiological studies that monitor the pathogen infiltration status among livestock animals, many people are concerned about the potential reputational damage to livestock producers that may be imposed by these studies. We need to manage such conflict of interests and develop cooperative relationships between stakeholders, including veterinarians, hunters, breeders, and farmers, which will enable sustainable and effective surveillance. Measures such as risk communication, informed consent, and the formulation of rules for handling samples are required to solve the issues that currently hinder epidemiological studies in animals.

From an international perspective, concerns about biological resources and dual use were also mentioned during the interviews. Access and Benefit-Sharing (ABS), a system under public international law that aims to fairly distribute benefits arising from genetic resources, regulates transboundary movement of biological samples on zoonosis research. While complying with ABS, it is necessary to take care so that sample movement between nations or data collection by local researchers can be carried out smoothly.

## 4 Conclusion

Here we conducted a thematic analysis of key-informant interviews with Japanese experts and a literature review of the OH approach, and two key themes were identified: types of required R&D and the systems needed to support them. These challenges may be common throughout the world, and this review attempted to integrate the experts' perspectives as a convergence of knowledge in an effort realize an ideal OH approach. Although this review is mainly focused on viral zoonoses, these insights are expected to contribute to other topics such as global threat of bacterial antimicrobial resistance. Researchers, policy makers and health officials are encouraged to incorporate these two themes to successfully design and implement OH projects in order to create a resilient society against future pandemics.

The following are the supplementary data related to this article.

Supplementary Table 1 List of interviewees Supplementary Table 1

Supplementary material: Interview procedure Image 1

## Declaration of Competing Interest

The authors declare that they have no competing interests.

## Data Availability

No data was used for the research described in the article.
