# Supplementary material for: Current research and future directions for realizing the ideal One-Health approach: A summary of key-informant interviews in Japan and a literature review
Source: One Health. 2022 Dec 5;16:100468. doi: 10.1016/j.onehlt.2022.100468 (PMC9721418; doi:10.1016/j.onehlt.2022.100468)
Supplement: Supplementary file 2 — Supplementary material: Interview procedure [file mmc2.docx]

**Supplemental document:** Interview procedure

The interviews started by asking participants to envision a society in 2050 which will be resilient to pandemics. Three semi-structured questions were asked; (1) how we can build such a society, (2) what innovations are required to accomplish this task, and (3) how the participant and their field could contribute to this. Depending on how they responded, different lines of prompts were given to explore their views on the participant’s current research or attempt relevant to One-Health, required technological innovations, and institutional barriers that may hinder interdisciplinary research. After each interview, the interview was immediately transcribed and key themes/words were identified. These themes and words were then explored at the following interviews and through literature reviews. This process was iteratively conducted until data saturation.
